# Toward an Attentive Robotic Architecture: Learning-Based Mutual Gaze Estimation in Human–Robot Interaction

**DOI:** 10.3389/frobt.2022.770165

**Published:** 2022-03-07

**Authors:** Maria Lombardi, Elisa Maiettini, Davide De Tommaso, Agnieszka Wykowska, Lorenzo Natale

**Affiliations:** ^1^ Humanoid Sensing and Perception, Istituto Italiano di Tecnologia, Genova, Italy; ^2^ Social Cognition in Human-Robot Interaction, Istituto Italiano di Tecnologia, Genova, Italy

**Keywords:** mutual gaze, joint attention, human–robot interaction, humanoid robot, computer vision, experimental psychology, attentive architecture

## Abstract

Social robotics is an emerging field that is expected to grow rapidly in the near future. In fact, it is increasingly more frequent to have robots that operate in close proximity with humans or even collaborate with them in joint tasks. In this context, the investigation of how to endow a humanoid robot with social behavioral skills typical of human–human interactions is still an open problem. Among the countless social cues needed to establish a natural social attunement, this article reports our research toward the implementation of a mechanism for estimating the gaze direction, focusing in particular on mutual gaze as a fundamental social cue in face-to-face interactions. We propose a learning-based framework to automatically detect eye contact events in online interactions with human partners. The proposed solution achieved high performance both *in silico* and in experimental scenarios. Our work is expected to be the first step toward an attentive architecture able to endorse scenarios in which the robots are perceived as social partners.

## 1 Introduction

Joint attention (or shared attention) is one of the most important mechanisms occurring in a non-verbal interaction between two or more individuals. It is achieved when individuals direct their gaze on the same object or event in the environment as a consequence of social gestures (e.g., gaze shift, pointing, and facial expressions) (Moore et al., 2014). The ability to establish joint attention is crucial in many mechanisms of social cognition, for example, comprehension, language development, and intention (Tomasello, 1995; Tomasello et al., 2005; Mundy et al., 2007). A failure in such abilities, indeed, represents one of the earliest and basic social impairments in autism and communicative deficits (Mundy and Neal, 2000; Dawson et al., 2004).

In this context, designing and building an attention architecture enabling joint attention between a human and an embodied artificial agent, such as iCub, has inspired many researchers from different fields, spanning from artificial intelligence to robotics and from neuro and cognitive science to social science (Henschel et al., 2020; Wykowska, 2020). Inspired by the behavior of human beings, our ambitious goal is to develop a robotic visual attention system that responds to several social cues characterizing an effective non-verbal human interaction. For example, as a social cue, eye gaze estimation plays a crucial role for the prediction of human attention and intention, and hence is indispensable for better understanding human activities (Kleinke, 1986; Emery, 2000). Humans, indeed, tend to look at an object before trying to grasp it with the hand (Voudouris et al., 2018). This implies that it is possible to predict human intention just observing where their attention is focused at.

In our long-range aim, the humanoid robot iCub will be able to establish social attunement with the human partner recognizing and reproducing a wide range of social abilities in a human-like manner. The robot’s ability to imitate human-like behaviors might bring the humans to adopt the so-called *intentional stance* as a strategy toward the robot like they do with other humans (Marchesi et al., 2019). As proposed by the philosopher Daniel Dennett, an intentional stance is the strategy of prediction and explanation that attributes beliefs, desires, and intentions to an agent and predicts its future behavior from what it would be rational for an agent to do given those mental states (Dennett, 1971).

In this research report, we present our first successful step in the ongoing implementation of such a robotic system. Specifically, we spent our initial effort on endowing iCub with the key ability of recognizing eye contact events. The report is organized in the following way. In the next section (Section 2), we discuss the importance of the mutual gaze in dyadic interactions. In Section 3, we describe the proposed solution for eye contact detection. We benchmarked this algorithm in Section 4 where we compare it against the state-of-the-art method. In Section 5, we test our architecture in a real HRI experimental setup, discussing the advantages of our solution in regard to the chosen case study. Finally, we draw the conclusion in Section 6.

## 2 Focus on Mutual Gaze and Motivation

In the context of joint attention, eye contact provides a foundation of effective social interaction since it signals the readiness for interaction and the attention of the partner. Given the sensitivity of a human when being watched by another one, it is not surprising that the mutual eye contact may influence the efficiency of the person-construal process (Macrae et al., 2002). For example, studies revealed that human observers are faster to detect target faces/eyes with a direct gaze than those with averted gaze (Coelho et al., 2006), and the perceived eye contact enhances the activation of components of the social brain network (Senju and Johnson, 2009).

While the effect of mutual eye gaze has been largely studied in human–human and human–screen scenarios with the use of reaction time measures (Galfano et al., 2012), saccadic behavior (Ueda et al., 2014; Dalmaso et al., 2017a; Dalmaso et al., 2017b), and EEG (Hietanen et al., 2008; Pönkänen et al., 2011), few works exist in the literature investigating whether similar attention mechanisms arise in human–robot scenarios as well (Boucher et al., 2012).

For example, in the context of human–human interaction, Chong et al. (2020) proposed a novel approach based on deep neural networks to detect eye contact using PoV cameras with reliability equivalent to expert human raters. The proposed algorithm has been used in this work as the baseline for the comparison (see Section 4.3).

Wykowska (2021) underlined the importance of the role of humanoid robots as a physical presence in real-time interaction since they provide higher ecological validity than screen-based stimuli and better experimental control than human–human interaction. Along the same line, Kompatsiari et al. (2018) exploited the widely used Posner paradigm (Posner, 1980) to propose a novel interactive protocol involving the humanoid robot iCub (Metta et al., 2010) and examine the impact of mutual gazes on the mechanisms of joint attention.

The Posner paradigm (together with its variations) is a neuropsychological test typically used to investigate attention orienting in response to a directional cue. In such a gaze-cueing task, the observer is typically asked to discriminate an object target (usually presented in a lateral location) while looking at a directional cue (e.g., schematic faces or arrows) presented centrally, in between the locations of potential target presentation. The cue can be either valid or invalid, depending on whether it pointed to the target object or to a different direction.

In their study, iCub was positioned between two lateral screens on which the target object was presented (in line with the Posner paradigm). iCub was used as the experimental apparatus both to establish a real-time eye contact with the human participant and to manipulate the directional gaze cue across the trials. The results revealed that the human reaction times depended on the combined effect of cue validity related to the iCub’s gaze direction and social aspect of mutual gaze. Another example can be found in Stanton and Stevens et al. (2017) where the Nao humanoid robot^1^ was used to study the impact of three different levels of a robot’s gaze (averted, constant, and situational) in cooperative visual tracking task. Nevertheless the main drawback of the aforementioned studies was the use of the robot as a passive stimulus. Specifically, in both studies, the humanoid robot was operated either with pre-programmed default text-to-speech and timed head movements or through pre-programmed gaze behavior. As such, the robot had neither any perception of a real human’s gaze nor any feedback from the surrounding environment.

Some authors support the notion that a robot embodying artificial models capable of reproducing human skills is a unique and invaluable tool to explain human cognition (Wainer et al. (2006); Pfeifer et al. (2007); Wykowska (2021)). With this motivation, in this work, we propose a new module for iCub which allows to automatically detect whether a mutual gaze is established with the human partner during the interaction. Specifically, the report consists of three main contributions:

1. *Dataset collection for mutual gaze detection in frontal human–robot interaction.* In the context of frontal tasks, the dataset collected is general enough to suit many different experimental scenarios. To the best of our knowledge, it is the first mutual-gaze dataset collected involving a humanoid robot.
2. *Designing, implementation, and training of a learning module based on the aforementioned dataset.* Such a module is then embedded into the iCub’s framework and validated both *in silico* and in online scenarios. Furthermore, we compared our method with the solution proposed in Chong et al. (2020), achieving an improvement in the accuracy of around 15 percentage points.
3. As a case study, we select the experimental setup proposed in Kompatsiari et al. (2018) where iCub was used as a passive experimental apparatus. Within this framework, we performed several controlled experimental trials to test our application in a time-constrained social robotic experiment.

Our approach aims at reducing the amount of hardware equipment required by the robot to detect a mutual gaze with the human partner (e.g., external cameras and eye tracker). The robot, indeed, relies only on the image frames captured by its eye-like cameras making the interaction as natural as possible. The algorithm developed in this work is an important building block for robotic setups that can be used to study human social cognition in naturalistic interactions.

## 3 Eye Contact Learning Approach

### 3.1 Data Collection

#### 3.1.1 Participants

A total of 24 participants were recruited for data collection (mean age = 29.54 ± 3.14, 15 women). All participants had normal or corrected normal vision (6 participants out 24 wore glasses) and provided written informed consent. The data collection was conducted at the Istituto Italiano di Tecnologia, Genoa, and it was approved by the Local Ethical Committee (Comitato Etico Regione Liguria).

#### 3.1.2 Setup

**FIGURE 1:**
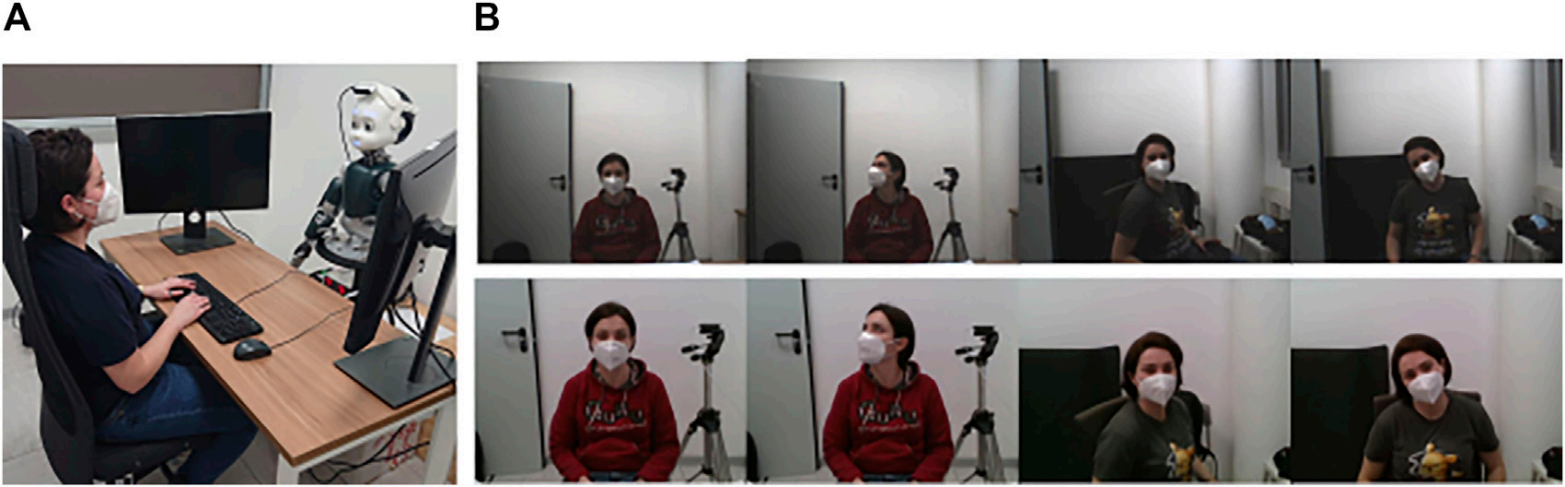
Dataset collection. **(A)** Overall setup. The participant was seated at a desk in front of iCub. The latter was mounted with a RealSense camera on its head. **(B)** Sample frames were recorded using both iCub’s camera (first row) and the RealSense camera (second row). Different frames capture different human positions (rotation of the torso/head) and conditions (eye contact and no eye contact).

The humanoid robot iCub embeds two Dragonfly2 cameras^2^ (right and left eyes); only one eye camera was used with the frame resolution set to 640 × 480 pixels. In this study, we used the right eye camera, but the left eye camera can also be used equivalently. In order to have higher quality images for the training phase of the proposed eye contact classifier, a second dataset was also collected using the Intel RealSense depth camera D435.^3^ (See Figure 1 for a visual evidence.) The RealSense camera was mounted on the iCub’s head through a 3D printed mount. The middleware YARP (Yet Another Robot Platform) (Metta et al., 2006) was used to integrate different modules (e.g., iCub’s controller, cameras, data dumper, and code modules). The recording setup is shown in Figure 1. In line with what we claimed in Section 1— that is, to avoid the need of external hardware—we underline that the RealSense camera was used only for acquiring training data. In the deployment phase, the system was always tested using images provided by the cameras in the eyes of the iCub.

#### 3.1.3 Task

Participants were asked to sit in front of the iCub at a distance of around 1 m and to establish first mutual gaze and then averted gaze with the iCub’s eyes in order to acquire frames both in eye contact and in no eye contact condition. In the eye contact recording session, participants were also asked to look at the iCub’s eyes but moving first their torso and then their head (Figure 1). For each position, the frame was captured both by the iCub’s right camera and the RealSense camera by pressing the bar space of the laptop’s keyboard. The final datasets consisted of 484 frames each (207 in eye contact and 277 in no eye contact conditions).

### 3.2 Eye Contact Classifier

Once the dataset was collected, the vector feature was extracted from each image by means of OpenPose^4^ (Cao et al., 2019), a well-known real-time system for multi-human pose estimation. Specifically, OpenPose takes *w* × *h* color image as input and produces the 2D locations (*x*, *y*) of anatomical keypoints for each person in the scene with the corresponding detection confidence level *k* as output. Relying on a multi-stage deep convolutional neural network, OpenPose can jointly detect body, face, hands, and foot keypoints reaching highly accurate and real-time performance, regardless of the number of people in the image.

In our work, a subset of 19 face keypoints were considered (8 points for each eye, 2 points for the ears, and 1 for the nose), resulting in a vector of 57 elements (i.e., the triplet (*x*, *y*, *k*) was taken for each point). Then, the detected keypoints were centered with respect to the head’s centroid, computed as the mean coordinates of all keypoints of the face, and normalized on the farthest point from the head’s centroid. The use of the face’s keypoints as a feature vector has the main advantage of making the classifier independent of the light conditions and the picture’s background.

The resulting feature vector is finally used as input to the binary classifier. A support vector machine (SVM) with the RBF kernel was chosen to address this classification task. We compared the results produced by the SVM with a random forest classifier; the former was chosen because it reported the best performance in terms of accuracy and F1 scores. (For a detailed comparison, see the Supplementary Material.) Moreover, given the results of the principal components analysis (PCA), we considered the RBF kernel. (See the Supplementary Material for further details.) The hyper parameters of the SVM model were selected using an exhaustive search over a grid of parameters and optimized by a 5-fold cross-validation (Pedregosa et al., 2011). After the training, the classifier’s output was the pair (*r*, *c*) where *r* = 1 if a mutual gaze is detected (0 otherwise), while $c\in \left[0,1\right]$ is the confidence level of the prediction.

**FIGURE 2:**
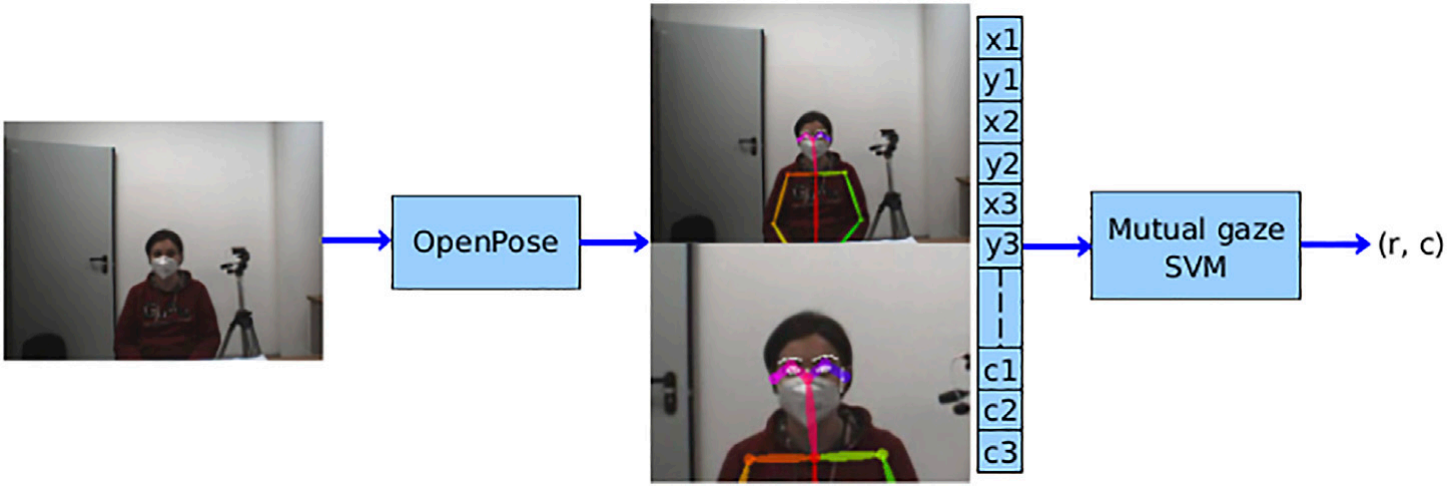
Learning architecture. The acquired image is first used as input for OpenPose in order to get the facial keypoints and build the feature vector for the individual in the scene. Then, such a feature vector goes in as input to the mutual gaze classifier whose output is the pair (*r*, *c*), where *r* is the binary result of the classification (eye contact/no eye contact) and *c* is the confidence level.

The overall learning architecture is depicted in Figure 2.

### 3.3 Training Details

The mutual-gaze classifier was trained both using the dataset collected with the RealSense and iCub’s eye. From now on, we refer to the classifier trained with the dataset *via* the iCub’s right eye since it reported higher performance metrics. (For a full comparison between the two datasets, see the Supplementary Material.)

The acquired dataset was augmented in order to be robust to the degenerative case in which OpenPose fails to detect the eyes’ boundaries and the pupils. To simulate such a condition, the coordinates of those keypoints in case of eye contact were set to zero, while the others (namely, the ones for nose, ears, and eyes) were left unchanged. Moreover, we applied a further augmentation by geometrically rotating the face’s keypoints, extracted by using OpenPose, to the left and right of a certain angle around the face’s centroid to cover a wider range of head rotations (not covered by the acquired samples). In detail, the facial keypoints were rotated to the left and right by an angle *α* ∈ {15°, 30°, 45°, 60°} taking the {5*%*, 10*%*, 10*%*, 5*%*} of the data, respectively. The final augmented dataset consisted of 654 samples (377 in eye contact and 277 in no eye contact conditions).

We handled the unbalanced dataset by properly weighing each class of classification. Such weights were chosen inversely proportional to class frequencies in the input data.

Finally, OpenPose parameters were tuned in order to have the best performance for the considered dataset (e.g., neural network resolution and images at different scales).

## 4 Results

### 4.1 Evaluation on the Collected Test Set

For the training of the classifier, the dataset was split into two subsets taking 19 out of 24 participants for the training set and the others 5 participants for the test set. The dataset was split *k* = 5 times in order to average the performance over different participant subsets and evaluate the statistical properties of the method. The performance was evaluated in terms of accuracy, precision, recall, and F1 scores reaching in all metrics values around 90*%*. Precisely we had accuracy = 0.91 ± 0.03, precision = 0.90 ± 0.08, recall = 0.89 ± 0.06, and F1-score = 0.89 ± 0.04.

### 4.2 Evaluation on Temporal Sequences

The mutual-gaze classifier was validated also on video streams recorded from the iCub’s camera during different controlled interactions with a human. In detail, four video streams were recorded in order to cover the following scenarios: 1) no mutual gaze, 2) frontal mutual gaze, 3) human rotating the head to left/right while maintaining a mutual gaze with the robot, and 4) human rotating the torso while maintaining a mutual gaze with the robot. To avoid the flickering in the classifier predictions caused by the high video frame rates, we implemented a mechanism to propagate the predictions to those frames for which the classifier output is not available due to frame rate incompatibilities. The reason behind this is that, in practical settings, it is reasonable to assume coherent predictions in a time span of ∼ 100 *ms*. To this aim, we implemented a buffer of 3 elements at the inference time. The actual classifier result was selected through a majority rule evaluated on the buffer. The implementation of the buffer allowed us to reach even a higher level of accuracy. Specifically, the accuracy registered in the first three scenarios reached its maximum value—that is, 1.0—, whereas in the last one the accuracy was 0.93. Analyzing the last scenario, we found that the classifier made wrong predictions when the human’s torso reached the extreme angles of 90 (right) and −90 (left) while keeping the head straight toward the robot (see the videos in the Supplementary Material). Such a drop in performance for extreme torso rotations is reasonable since the classifier was trained only for the frontal task.

### 4.3 Comparison With State-of-the-Art Method

In this section, the mutual gaze classifier is compared with the solution proposed in Chong et al. (2020). To the best of our knowledge, this is the most recent solution in the current literature that best adapts to our purposes. In Chong et al. (2020), the authors trained a deep convolution neural network (i.e., ResNet-50 (He et al., 2016)) as the backbone to automatically detect eye contact during face-to-face interactions. As network performance, the authors reported an overall precision of 0.94 and an F1-score of 0.94 on 18 validation subjects. The network was trained only with egocentric cropped frames of the individuals’ face.

Because the training code of Chong et al. (2020) was not released by the authors, we used the publicly available pre-trained model. We tested this model on our scenario where the participants wore face masks due to COVID-19’s ordinance, and the frames captured by the robot were low quality frames. Since the algorithm used in Chong et al. (2020) failed to detect the bounding boxes of the humans’ face in 33*%* of cases (probably due to the face masks), we used OpenPose for the bounding box detection. Such a bounding box was then used to crop the image sent as input to the convolution neural network. This was done to obtain a fair comparison between the two algorithms. The accuracy and F1 score were evaluated as metrics both on the test set and on the video streams.

• **Proposed approach**
  • *Test set.* Accuracy = 0.91 ± 0.03; F1 score = 0.89 ± 0.04.
  • *Stream videos.* Accuracy = 0.97; F1 score = 0.98.
• **Chong et al. (2020)** **+** **OpenPose**
  • *Test set.* Accuracy = 0.76 ± 0.05; F1 score = 0.77 ± 0.06.
  • *Stream videos.* Accuracy = 0.89; F1 score = 0.82.

Since data were normally distributed (Shapiro–Wilk test, *p*-value >0.05 ), the paired *t*-test was performed to assess the statistical difference between the performance of the two approaches (accuracy: *p*-value = 0.01, Cohen’s d = 2.009, 95*%* CI for Cohen’s d [0.385, 3.581]; F1 score: *p*-value = 0.037, Cohen’s d = 1.375, 95*%* CI for Cohen’s d [0.072, 2.609]).

On the test set, we obtained an improvement of 15*%* in the accuracy and of 12*%* in the F1 score, whereas on the video streams, we obtained an improvement of 8*%* in the accuracy and of 6*%* in the F1 score. In addition, our method was based on a low dimensional feature vector computed from facial and body landmarks. With respect to Chong et al. (2020) and other methods based on RGB information, it can be trained with less expensive hardware and without acquiring sensitive information (i.e., full RGB images depicting faces) from subjects.

The drop in the performance reported by Chong et al. (2020) in their work demonstrates the need of collecting a new dataset and shows that the current approaches in the literature are not suitable for our scenario. Indeed, the considered setting is challenging both for the presence of face masks and for the low-resolution camera that is often available in humanoid robots. On the contrary, Chong et al. (2020) used high-resolution cameras from camera glasses (1080p resolution). Notably, we could not compute the performance of our algorithm on the dataset used in Chong et al. (2020) because the latter was not made publicly available due to constraints imposed by the IRB protocol.

### 4.4 Model Interpretability

With the aim of understanding which face keypoints have larger contribution to the final output of the learning architecture, SHAP analysis was performed on the trained SVM model. SHAP (SHapley Additive exPlainations) is a method based on the coalitional game theory used to explain individually how each prediction is made by the learning algorithm. For each individual prediction, a value (SHAP value) is assigned to each feature as the measure of its impact on the model’s output. The final contribution for each feature is evaluated by averaging its SHAP values over a set of predictions (Lundberg and Lee, 2017).

**FIGURE 3:**
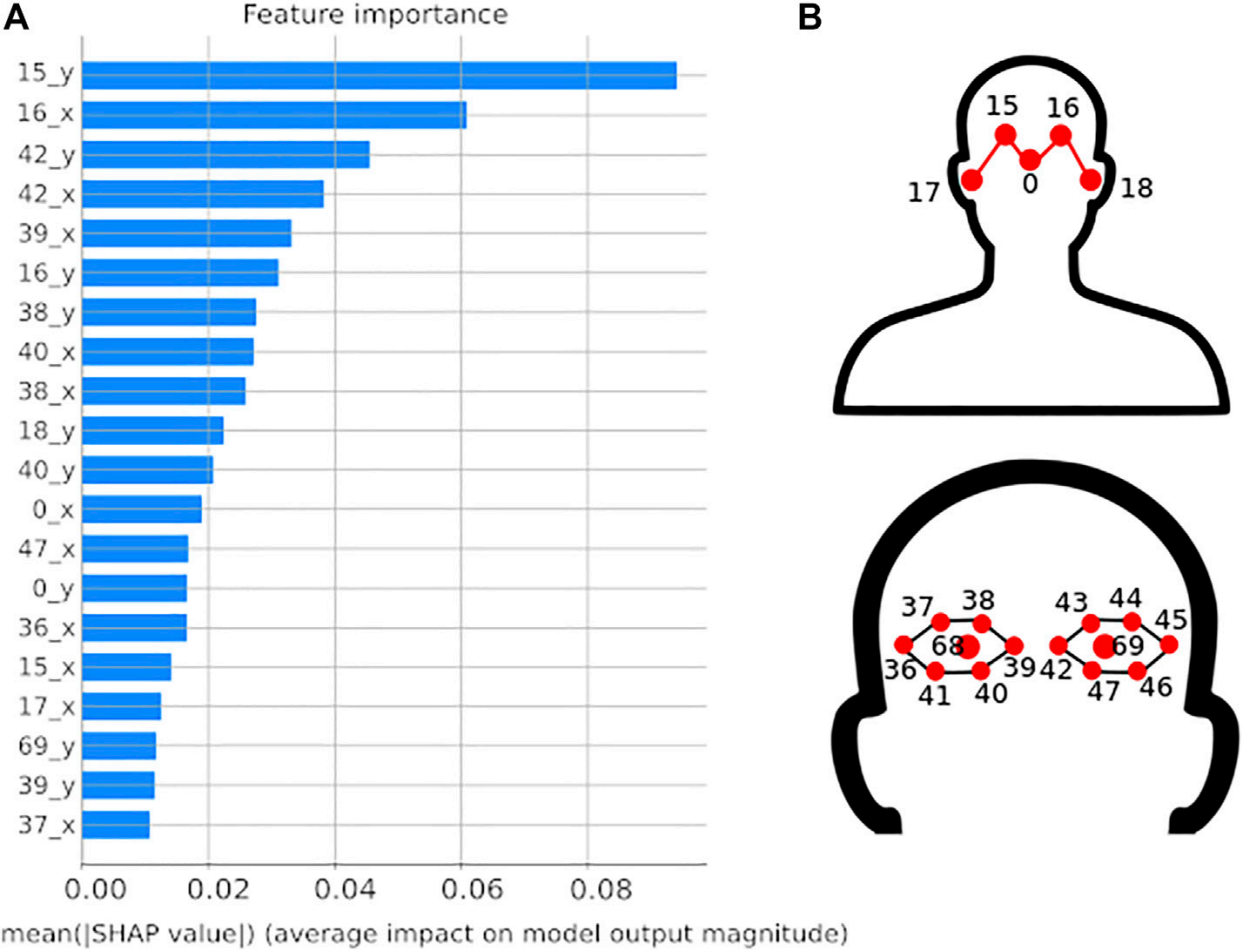
Feature importance. **(A)** Bar plot reporting on the *x*-axis the SHAP feature importance in percentage measured as the mean absolute Shapley value. Only the first 20 most important features are reported on the *y*-axis. **(B)** Numbered face keypoints of the feature vector.

In Figure 3 the bar plot of the feature’s impact on the model output is reported for the first 20 most important face keypoints. It can be observed that the internal points of the eyes (points 15, 16, 38, 39, 40, and 42) and partially the ears (point 18) have a mean SHAP value between 0.02 and 0.09; this means that a change in these input features has an impact on the prediction of around 2 − 9*%* percentage points. The analysis revealed that there is no feature that predominate the others, but all the elements of the feature vector make a comparable contribution to the prediction of the output. This was also confirmed by principal component analysis (PCA) reported in the Supplementary Material. The PCA performed on the data, indeed, did not make any improvement to the system implying that none of the considered features was completely redundant.

## 5 Deployment in an Experimental Setup

**FIGURE 4:**
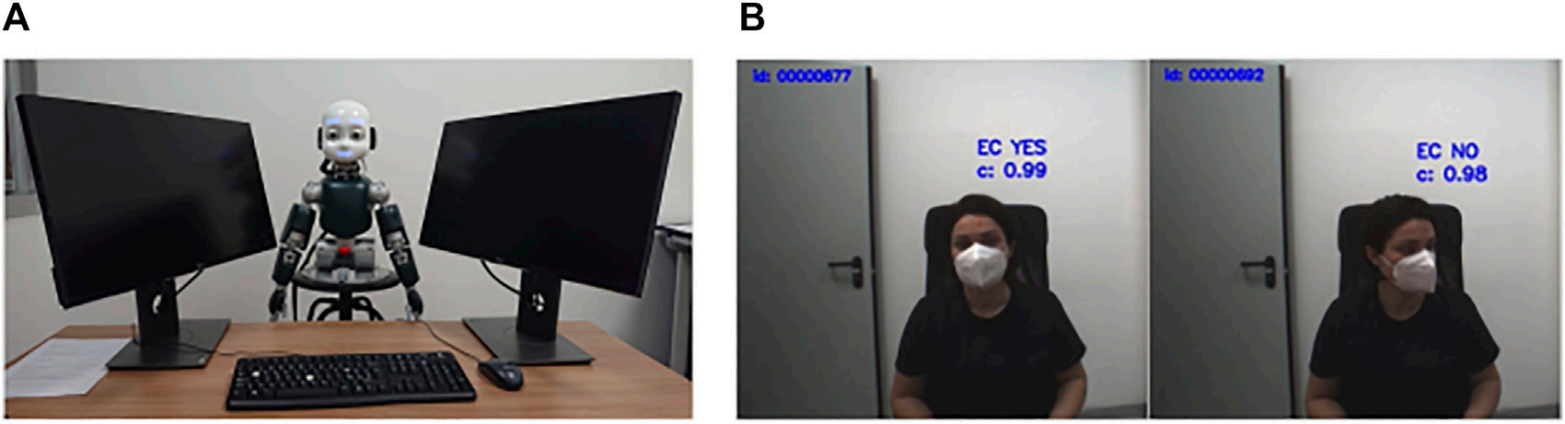
Experimental setup. **(A)** The iCub is positioned between two lateral screens face to face with the participant at the opposite sides of a desk that is 125 cm wide. **(B)** Sample frames acquired during the experiment in which the participant first looks at the robot to make an eye contact and then simulates a distraction looking at the lateral screen. On each frame, the prediction (eye contact yes/no) with the confidence value *c* is also reported.

Next, we further validated our approach presented in Section 3. As a test bed example, we integrated our algorithm in the experimental scenario presented in Kompatsiari et al. (2018). In such a setup, the participants were seated face to face with the iCub robot at a 125-cm-wide desk. iCub was positioned between two lateral screens on which target letters were presented to the participant. Also, iCub’s height was set at 124 cm from the floor in order to have its eyes aligned with participants’ eyes (Figure 4).

The conclusions of Kompatsiari et al. (2018) were based on the assumption that a mutual gaze was established between the subjects and robot, as confirmed by manual annotation by an experimenter. Therefore, the solution presented here offers a significant advancement as it provides an automatic mechanism that can avoid manual annotation and implements a contingent robot behavior allowing bidirectional eye contact mechanisms, which, as shown by the results of Kompatsiari et al. (2018), are crucial for establishing joint attention in HRI.

The experimental trial was designed as follows:

• iCub starts with the head pointing down and with its eyes closed for 2 s;
• it opens its eyes for 500 ms without moving the head;
• iCub looks toward the participant’s eyes (eye contact) for 2.5 s;
• iCub moves the head laterally toward one of the lateral screens, where the letter V or T appeared randomly either on the same screen where the robot is looking at (valid trial) or on the opposite screen (invalid trial) for 200 ms; and
• the participant was instructed to identify the target letter by pressing V or T on the keyboard while keeping a mutual gaze with the robot and without gazing at the screen.

To validate the classifier, we asked a total of 4 participants to carry out 8 blocks of 8 trials each. The experiments were controlled in order to have the ground truth for each block of trials. In detail, the participant was asked to maintain a mutual gaze with the robot in 5 blocks of trials and to always simulate a distracted participant in the other 4 blocks left (e.g., checking the phone and looking at the lateral screens). To assure the quality of the ground truth, the experimenter monitored online eye movements of the participants, and the trials were further checked offline before the analysis. Only one trial was discarded.

As done before, the performance was evaluated in terms of accuracy, precision, recall, and F1 scores. We registered accuracy = 0.97, precision = 0.95, recall = 1.00, and F1 score = 0.97.

## 6 Conclusion

In this research report, we presented our first results of an ongoing work aiming at developing a novel attentive architecture for the humanoid robot iCub. In this context, we focused on the social cue of the mutual gaze making iCub capable of recognizing eye contact events while interacting online with a human partner. We validated the proposed mutual gaze classifier both computationally and experimentally, showing high performance values. We also compared the proposed approach with the state-of-the-art method described in Chong et al. (2020), reporting a consistent improvement in the performance. We underline that our method requires neither any additional hardware (e.g., external camera and eye tracking glasses) nor a robot with embedded high-quality and expensive eye cameras. Another advantage of our method is that it uses relatively low dimensional features extracted by facial landmarks which are intrinsically anonymous. With respect to other methods that use RGB information, it can be re-trained with less expensive hardware and without storing personal data from the subjects. Our results may potentially allow the research community to use an active robotic framework in more complex interactive scenarios helping the study of human cognition. For example, it has been previously found that the mutual gaze condition increases the level of engagement and/or is rewarding during a human–robot interaction compared to an averted gaze (Kampe et al., 2001). Similarly, Schilbach et al. (2010) investigated the neural correlation of joint attention finding that following or directing someone else’s gaze activates several cortex areas of the brain related to the coordination of perceptual and cognitive processes.

Improving and extending the mutual gaze scenario to a wider problem of the gaze estimation is a part of our current research. As a potential improvement, temporal information (e.g., temporal coherence between consecutive frames, and optical flow) from dynamic data, such as videos, could bring additional information to the system increasing performance and generalization capabilities. Furthermore, the implementation of an attention system with the ability to detect social cues is a fundamental step toward the realization of socially capable humanoid robots.

## Data Availability

The anonymised data that support this study, the code and the learning trained models can be found at https://github.com/hsp-iit/mutual-gaze-detection.git. Further inquiries can be directed to the corresponding author.

## Footnotes

1
https://www.softbankrobotics.com/emea/en/nao.

2
http://wiki.icub.org/images/c/c9/POINTGREY_-_Dragonfly2.pdf.

3
https://www.intelRealSense.com/depth-camera-d435/.

4
https://github.com/CMU-Perceptual-Computing-Lab/openpose, https://github.com/robotology/human-sensing.
