# Supplementary material for: Toward an Attentive Robotic Architecture: Learning-Based Mutual Gaze Estimation in Human–Robot Interaction
Source: Front Robot AI. 2022 Mar 7;9:770165. doi: 10.3389/frobt.2022.770165 (PMC8935014; doi:10.3389/frobt.2022.770165)
Supplement: Supplementary file 1 [file DataSheet1.pdf]

Supplementary Material

The proposed approach resulted from a series of comparisons between different configurations. Specifically, we carried out an analysis considering first the use of the Principal Component Analysis (PCA) and then random forest as an alternative algorithm for the detection of mutual-gaze condition. Specifically, Section 1 is dedicated to the principal component analysis while Section 2 shows the comparison between the SVM and the random forest classifier with and without the PCA. Furthermore, we compared the SVM classifier trained with the dataset acquired both from the iCub's camera and from the Intel RealSense camera (Section 3). In Section 4 we report further details on the baseline we used to compare our solution with the current state-of-the-art method. Finally, an analysis on the confidence scores estimated by the mutual gaze classifier is reported in Section 5.

1 PRINCIPAL COMPONENT ANALYSIS

Because the OpenPose keypoints considered as feature vector seemed to be redundant, the PCA was performed to reduce their dimensionality. Figure S1(a) shows the cumulative plot of the explained variance of the collected dataset. As we can see from the plot, only 6 principal components are required to explained more than 95% of the total variance in the data. In Figure S1(b-c) the transformed data are plotted both using the first two and the first three principal components. The two learning classes (eye contact, no eye contact) can be grouped into well-defined clusters. Specifically, the no eye contact class extends mainly on the third principal component whereas the eye contact class on the first two components.

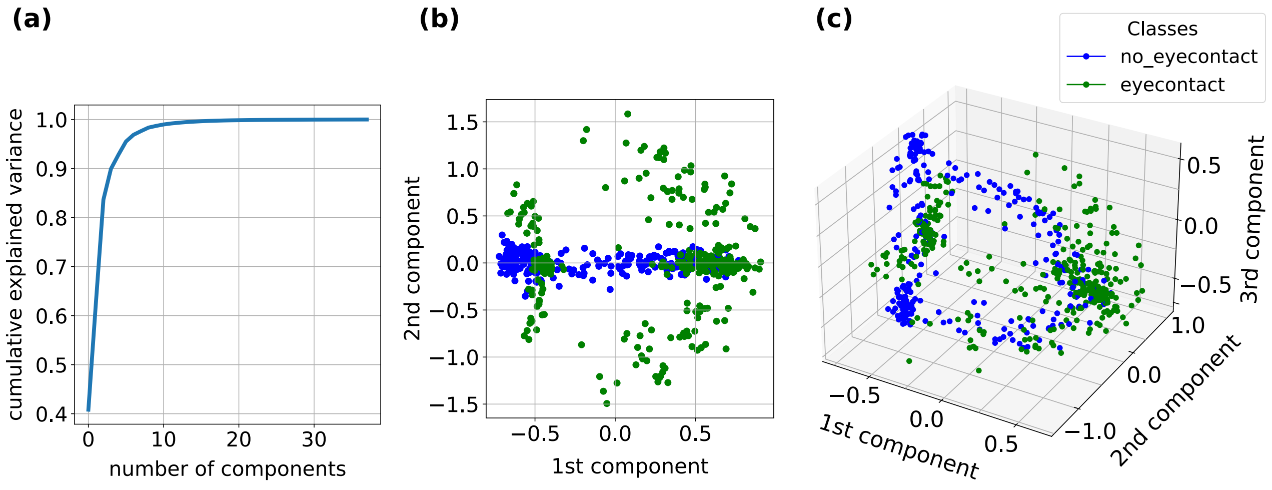

Figure S1. Principal component analysis. (a) Cumulative plot of the explained variance of the data over the number of principal components. **(b-c)** 2D and 3D scatter plot of the data used for the training of the classifier considering two and three principal components respectively. The class *eye contact* is in green whereas the class *no eye contact* is in blue.

2 COMPARISON BETWEEN THE SVM AND THE RANDOM FOREST ALGORITHM

In this Section, we make a comparison between the support vector machine and the random forest algorithms using as training samples either raw from OpenPose or the components extracted with the PCA. Both algorithms were trained and tested on the same train and test set (train set: 19 out of 24 participants, test

set: 5 participants). The train set was augmented rotating geometrically the input features to the left and right as described in the main article. The performance measured in terms of accuracy, precision, recall and F1 score are reported in Table S1. As in Section 5 of the main article, we considered mean and standard deviation on $k = 5$ random splits of the dataset. The SVM classifier performs better than the random forest reaching values around 90% for all metrics. Furthermore, the SVM without the PCA reports mean values with narrow standard deviation with respect to those resulting after the PCA.

Table S1. Comparison SVM - Random forest. Mean and standard deviation of accuracy, precision, recall and F1 score are reported for the support vector machine and the random forest algorithm trained with the same dataset (that is the dataset collected with iCub's right eye). For each algorithm, the metrics are evaluated with and without the principal component analysis of the input features.

	**SVM**		**Random forest**	
	with PCA	*without PCA*	*with PCA*	*without PCA*
Accuracy	0.89 ± 0.04	$\mathbf{0.91 \pm 0.03}$	0.89 ± 0.04	0.88 ± 0.05
Precision	0.90 ± 0.06	$\mathbf{0.90 \pm 0.08}$	0.86 ± 0.08	0.87 ± 0.11
Recall	0.83 ± 0.13	$\mathbf{0.89 \pm 0.06}$	0.88 ± 0.07	0.86 ± 0.09
F1 score	0.86 ± 0.08	$\mathbf{0.89 \pm 0.04}$	0.87 ± 0.05	0.86 ± 0.07

3 COMPARISON BETWEEN THE ICUB'S CAMERA AND THE INTEL REALSENSE CAMERA

To determine the impact of a higher level of details in the input image, we compared the results obtained by using the datasets collected with the iCub's camera (Dragonfly 2) and the high quality Intel RealSense D435i camera. Results are reported in Table S2. They were evaluated considering mean and standard deviation of the metrics on $k = 5$ random splits of the dataset. The SVM was trained using both datasets individually and tested on the test set collected from the iCub's camera. This choice was motivated by the fact that during the deployment the algorithm is required to take the input only from the Dragonfly 2 to avoid external hardware that may influence the human behaviour during the interaction with the robot. Despite the high quality of the images coming from the Intel RealSense, the classifier trained with the dataset collected using the Dragonfly 2 performs better, especially in precision and F1-score metric where it reports also narrow standard deviations.

Table S2. Performance on iCub's test set. Mean and standard deviation of accuracy, precision, recall and F1 score are reported for the support vector machine algorithm trained both with the data acquired from the iCub's camera and with the data acquired from the Intel RealSense camera and tested on the data collected only from the iCub's eye.

	iCub Test set	
	RealSense Train set	*iCub Train set*
Accuracy	0.90 ± 0.05	$\mathbf{0.91 \pm 0.03}$
Precision	0.86 ± 0.06	$\mathbf{0.90 \pm 0.08}$
Recall	0.82 ± 0.18	$\mathbf{0.89 \pm 0.06}$
F1 score	0.83 ± 0.1	$\mathbf{0.89 \pm 0.04}$

For the sake of completeness, the algorithm was also tested on the test set collected from the RealSense both when it was trained on the train set collected from the RealSense and on the one from the iCub. The performances are reported in Table S3. We observe that the performance obtained using images from the RealSense camera (both for training and testing) is slightly better. That is completely reasonable due to the quality of the camera if compared to that one embedded in the iCub's eye. Nevertheless, the increase in performance is relatively minor and it does not justify the need for additional hardware.

Table S3. Performance on Intel RealSense test set. Mean and standard deviation of accuracy, precision, recall and F1 score are reported for the support vector machine algorithm trained both with the data acquired from the iCub's camera and with the data acquired from the Intel RealSense camera and tested on the data collected only from the RealSense camera.

	Realsense Test set	
	iCub Train set	*RealSense Train set*
Accuracy	0.9 ± 0.05	0.92 ± 0.03
Precision	0.84 ± 0.11	0.92 ± 0.04
Recall	0.89 ± 0.11	0.89 ± 0.07
F1 score	0.85 ± 0.07	0.90 ± 0.03

4 FURTHER DETAILS ON THE BENCHMARK

As discussed in the main article, we compared the presented approach with the state-of-the-art method proposed in Chong et al. (2020). In this Section, we report further details on how we compared the two algorithms. Briefly, for the baseline the bounding box of the human face in the picture is first detected by means of *dlib*, an open source C++ toolkit containing machine learning algorithms, and then the cropped image is sent to the convolutional neural network to estimate eye contact events. The bounding box detection based on dlib failed in 33% of cases probably due to the presence of facemasks in the dataset. To make a fair comparison, we replaced dlib with an algorithm to detect the bounding box of the faces based on OpenPose. For each frame, the baseline produces as output a score s ranging from 0 (no eye

contact) to 1 (eye contact). In the evaluation of the metrics, we considered the baseline's prediction as *eye contact* if $s \geq 0.5$ (*no eye contact* otherwise). The baseline output is depicted in Figure S2.

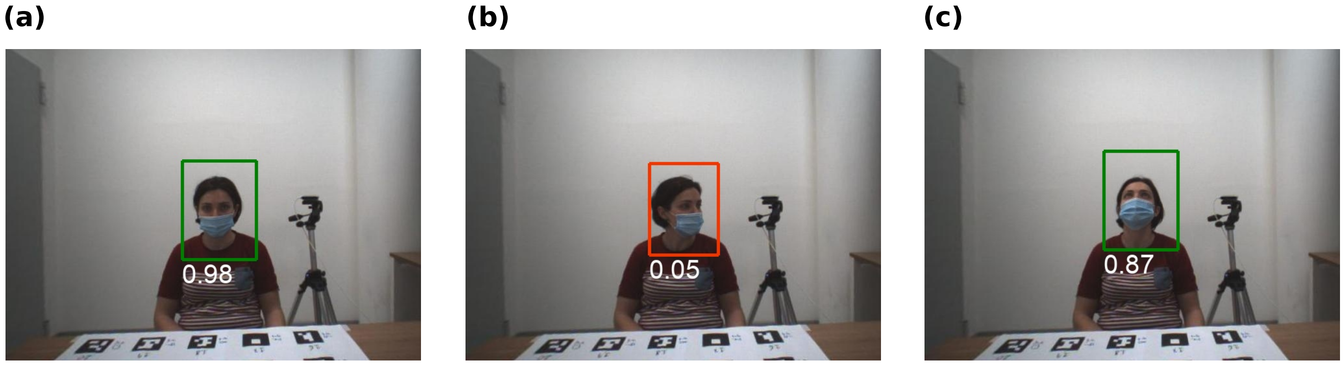

Figure S2. Baseline output. Sample frames representing the baseline output in three different cases: **(a)** right predicted eye contact, **(b)** right predicted no eye contact, **(c)** predicted eye contact in no eye contact event. The bounding box surrounding the human face is built by mean of OpenPose, while a score indicates the classifier's prediction. The colour of the bounding box varies from red (score $= 0.0$) to green score $= 1.0$.

5 DISCUSSION ON THE CONFIDENCE LEVEL REPORTED BY THE MUTUAL GAZE CLASSIFIER

In this Section we analyse the confidence level provided by the mutual gaze classifier when used in a natural scenario. To this aim, we registered the confidence scores for the video attached to the article as supplementary material. In Figure S3 we plot the confidence level frame-by-frame (from second 5 to second 61 of the video) marking the *eye contact* prediction with a solid blue line and the *no eye contact* prediction with a solid red line. We notice sharp drops in the confidence level during the switching between eye contact and no eye contact conditions. Specifically:

- the first switch (head oriented from frontal to right) is from frame 22 to frame 31 (seconds $7 - 9$ of the video);
- the second switch (head oriented from down to frontal) interests frames from 115 to 122 (seconds $18 - 20$ of the video);
- from frame 145 to frame 220 the confidence level assumes an oscillating behaviour (seconds $22 - 32$). This corresponds to the sequence of frames in which the participant keeps eye contact with the iCub while moving their torso towards left and right. The segments in red in this interval happen when the participant has their torso completely rotated to the right or to the left. We can observe that even if some frames were wrongly classified as *no eye contact* (frames $160 - 176$ and frames $203 - 206$), the classifier reported a lower confidence level if compared to the *no eye contact condition* where it assumed values close to 1 (from frame 30 to frame 115);
- from the frame 225 to 395 the confidence level keeps the oscillating behaviour (seconds $30 - 54$). This second oscillation corresponds to the sequence of frames in which the participant rotates their torso and then moves their head in order to establish eye contact with the iCub. Also in this case, the segments in red are when the participant has their torso completely rotated to the right or to the left;
- last switch occurs from frame 385 to frame 398 (seconds $53 - 55$) when the participant returns in a frontal position.

As general comment, the confidence scores during the transitions tend to be lower (< 0.9) than those at steady state (> 0.9). In Figure S4 the sequences of some events of transition are reported as example.

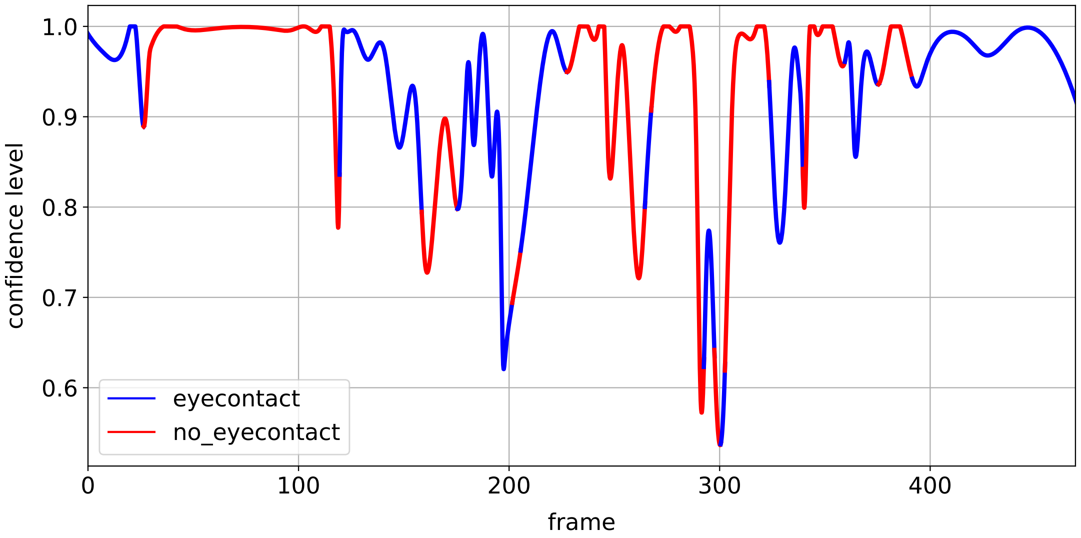

Figure S3. Confidence level. Confidence level (y-axis) registered for the video attached as supplementary material is reported frame-by-frame (x-axis). Solid blue line refers to eye contact condition whereas solid red line refers to no eye contact condition.

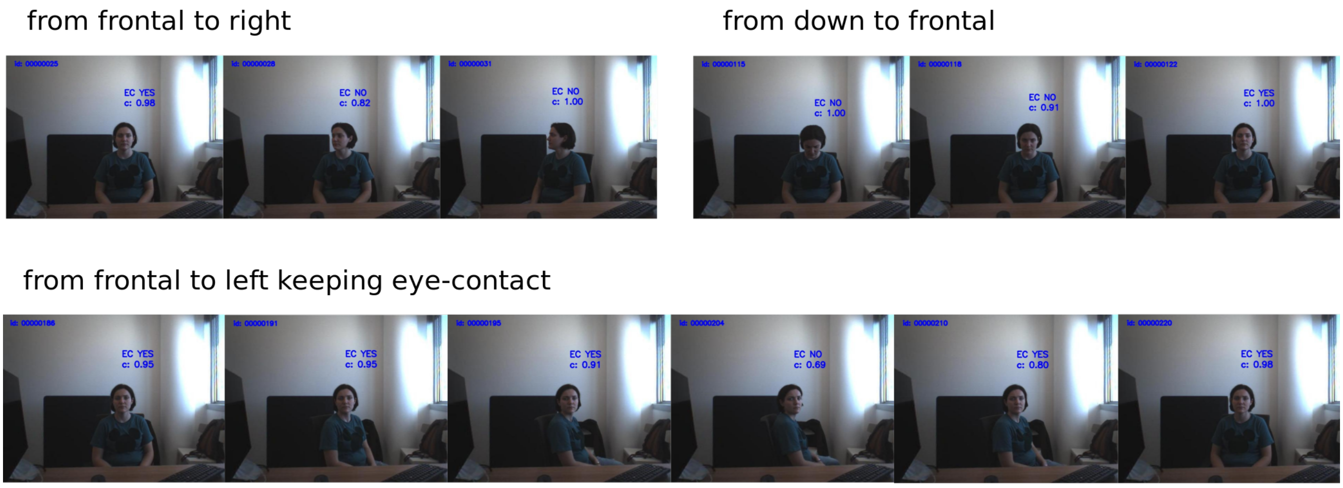

Figure S4. Transition scenarios. Three sequences of frames representing different transition scenarios: from frontal to right, from down to frontal and from frontal to left keeping eye-contact with iCub. The prediction with the confidence score are in blue on each single frame.

REFERENCES

Chong, E., Clark-Whitney, E., Southerland, A., Stubbs, E., Miller, C., Ajodan, E. L., et al. (2020). Detection of eye contact with deep neural networks is as accurate as human experts. *Nature communications* 11, 1–10
